# Vasculitis in Juvenile-Onset Systemic Lupus Erythematosus

**DOI:** 10.3389/fped.2019.00149

**Published:** 2019-05-09

**Authors:** Eve M. D. Smith, Hanna Lythgoe, Christian M. Hedrich

**Affiliations:** ^1^Department of Women and Children's Health, Institute of Translational Medicine, University of Liverpool, Liverpool, United Kingdom; ^2^Department of Paediatric Rheumatology, Alder Hey Children's NHS Foundation Trust, Liverpool, United Kingdom; ^3^St Helen's and Knowsley Teaching Hospital NHS Trust, St Helens, United Kingdom

**Keywords:** childhood lupus, JSLE, vasculitis, cutaneous vasculitis, visceral vasculitis

## Abstract

Juvenile-onset systemic lupus erythematosus (JSLE) is a rare, heterogeneous multisystem autoimmune disease that can affect any organ, and present with diverse clinical and serological manifestations. Vasculitis can be a feature of JSLE. It more commonly presents as cutaneous vasculitis than visceral vasculitis, which can affect the central nervous system, peripheral nervous system, lungs, gut, kidneys, heart, and large vessels. The incidence and prevalence of vasculitis in JSLE has not been well described to date. Symptoms of vasculitis can be non-specific and overlap with other features of JSLE, requiring careful consideration for the diagnosis to be achieved and promptly treated. Biopsies are often required to make a definitive diagnosis and differentiate JSLE related vasculitis from other manifestations of JSLE, vasculopathies, and JSLE related antiphospholipid syndrome. Visceral vasculitis can be life threatening, and its presence at the time of JSLE diagnosis is associated with permanent organ damage, which further highlights the importance of prompt recognition and treatment. This review will focus on the presentation, diagnosis, management and outcomes of vasculitis in JSLE, highlighting gaps in the current evidence base.

## Introduction

Vasculitis is a well-recognized feature of juvenile-onset systemic lupus erythematosus (JSLE). Despite this, there is a paucity of literature in this area. Vasculitis is defined by inflammatory changes to vessel walls, affecting different types (arteries, veins, capillaries) and/or sizes of vessels (large, medium, small) and a variety of sites (e.g., skin or visceral/internal organs). Symptoms of JSLE-related vasculitis can be non-specific (e.g., fatigue, fever, weight loss) and overlap with other features of the disease. Careful consideration is required to detect vasculitis, in particular visceral vasculitis, which is less common but potentially life-threatening and requires prompt and aggressive treatment. Histopathological assessment of tissue biopsies are the diagnostic gold standard, but patients may be classified with “probable vasculitis” based on clinical features alone (1). This review will update readers on literature available and highlight gaps in the current evidence-base.

Vasculitis can be associated with disease flares in SLE (2–4), and is therefore integral part of disease activity assessment tools, including the Systemic Lupus Activity Index (SLAM), British Isles Lupus Assessment Group (BILAG), and Systemic Lupus Activity Assessment Index 2000 (SLEDAI) (5). Patients with skin lesions that are not specific to SLE, such as cutaneous vasculitis, experience significantly more active disease when compared to patients with lupus-specific skin lesions only (e.g., malar rash) (3). The presence of major organ vasculitis at baseline influences the damage trajectory in JSLE patients, and is associated with greater JSLE related permanent organ damage (6).

## Cutaneous Vasculitis

### Epidemiology

The exact prevalence of cutaneous vasculitis in JSLE is not known. A study involving 179 patients with JSLE in the UK found that 12% experienced cutaneous vasculitis (7). A Brazilian study of 414 patients with SLE including 60 patients with JSLE found that 21.6% of JSLE patients developed cutaneous vasculitis compared with 15.4% of those with adult-onset disease (aSLE, difference was not statistically significant) (8). A study involving 50 aSLE patients found individuals with cutaneous vasculitis to be significantly younger when compared to patients who did not develop vasculitis (26.5 vs. 30.3 years; *p* = 0.018) (9).

### Associations With Other Organ Manifestations of SLE

In aSLE cutaneous vasculitis has been shown to be associated with lupus nephritis, hyopocomplementaemia (9, 10), musculoskeletal, constitutional, cardiovascular manifestations and Sjogren's syndrome (9). In a further study involving 170 aSLE patients, patients with lupus nephritis were shown to be at increased risk of cutaneous vasculitis (10). Lastly, cutaneous vasculitis may also be associated with neuropsychiatric lupus in aSLE (11, 12).

### Clinical Presentation and Pathophysiology

In JSLE, skin manifestations can be divided into lupus-specific (e.g., malar rash, discoid lupus, panniculitis) and lupus non-specific, including cutaneous vasculitis. SLE-associated cutaneous vasculitis affects small or medium-sized vessels in the skin and subcutaneous tissues. It has a wide variety of presentations that depend on the size of vessels involved and the extent of the vasculature affected. Cutaneous vasculitis most frequently affects the lower and upper limbs (13).

**Figure 1:**
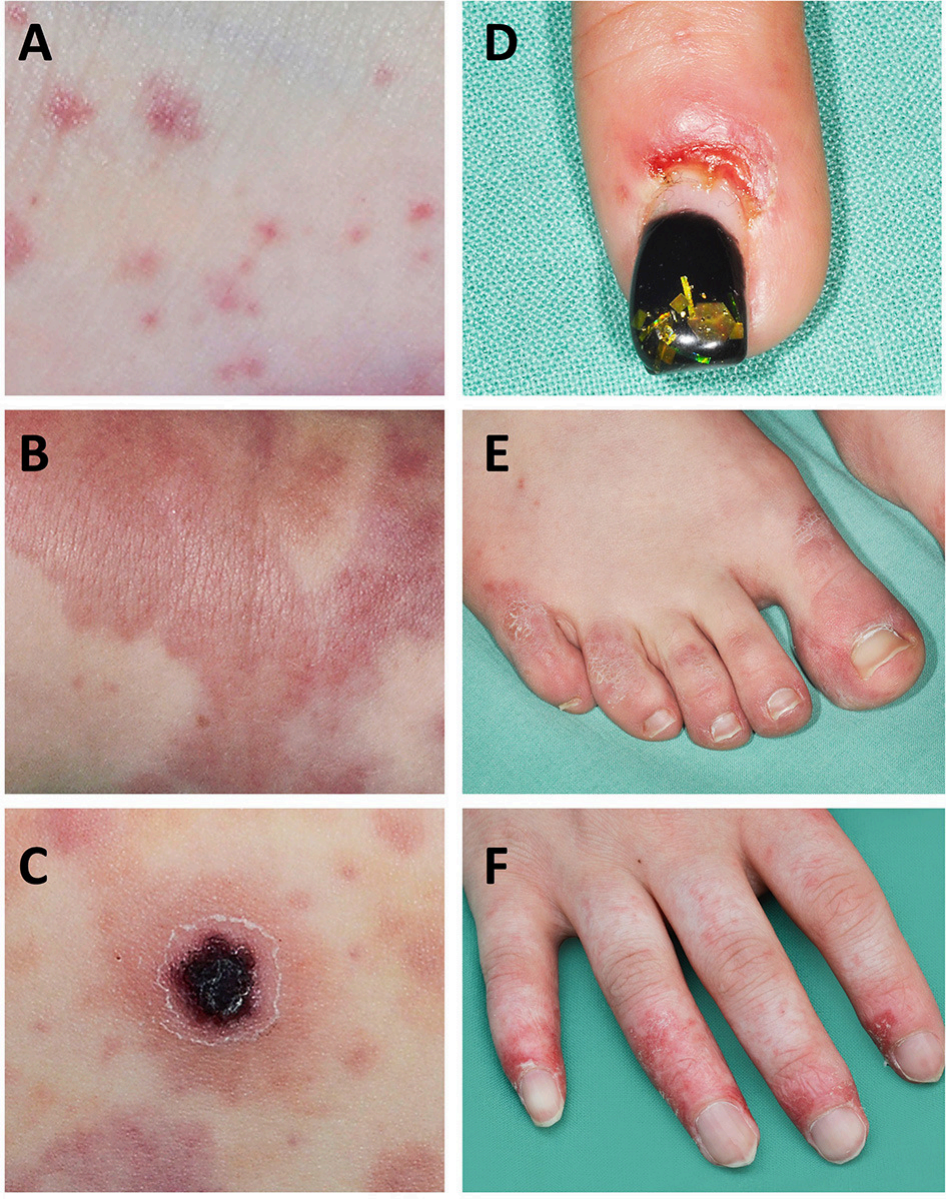
Skin manifestations in SLE and SLE-like disease. Small vessel vasculitis is a “common” feature in SLE-associated skin vasculitis. **(A)** Petechia and ecchymosis are the result of capillary inflammation and red blood cell extravasation; **(B)** palpable purpura are caused by inflammatory damage to venules and/or arterioles; **(C)** ulcerations and tissue necrosis are the result of reduced perfusion; **(D,E)** chilblain lesions can manifest as chilblains (cold induced sores) that may ulcerate, or painful and/or itchy bluish-reddish discoloration with swelling; **(F)** vasculopathy and finger atrophy in a patient with complement deficiency and secondary type I interferon upregulation.

Vasculitis affecting the **small vessels** of the skin (arterioles, capillaries, post-capillary venules in the superficial, and mid-dermis) usually presents with petechiae, purpura, and/or punctate vasculitis lesions. Petechiae are pinprick macules which do not blanch and are not palpable, resulting from capillary inflammation and red blood cell extravasation (Figure 1A) (14). Purpura are caused by inflammation of venules and/or arterioles and consist of larger papules and plaques which do not blanch and become palpable as damage progresses (Figure 1B) (14). Punctate vasculitic lesions, ulcerations and tissue necrosis are caused by reduced perfusion; shallow ulcers are caused when this affects the small vessels and deeper ulcers are caused when medium-sized vessels are affected (Figure 1C).

Vasculitis of **medium sized vessels** in the dermis or subcutaneous layers may cause livedo reticularis, nodules, and/or the aforementioned deep ulcers (15). Livedo reticularis is a small or widespread area of mottled, reticulated, reddish-purplish discoloration of the skin caused by compromised blood flow in the medium-sized vessels (15). Cutaneous ulcers, nodules, digital gangrene, livedo racemosa, and pyoderma-gangrenosum-like lesions are indicative of arterial involvement. Individuals affected have higher probability of associated visceral vasculitis (16). Lesions mimicking vasculitis can be caused by haemorrhagic and vaso-occlusive disease (17).

**Figure 2:**
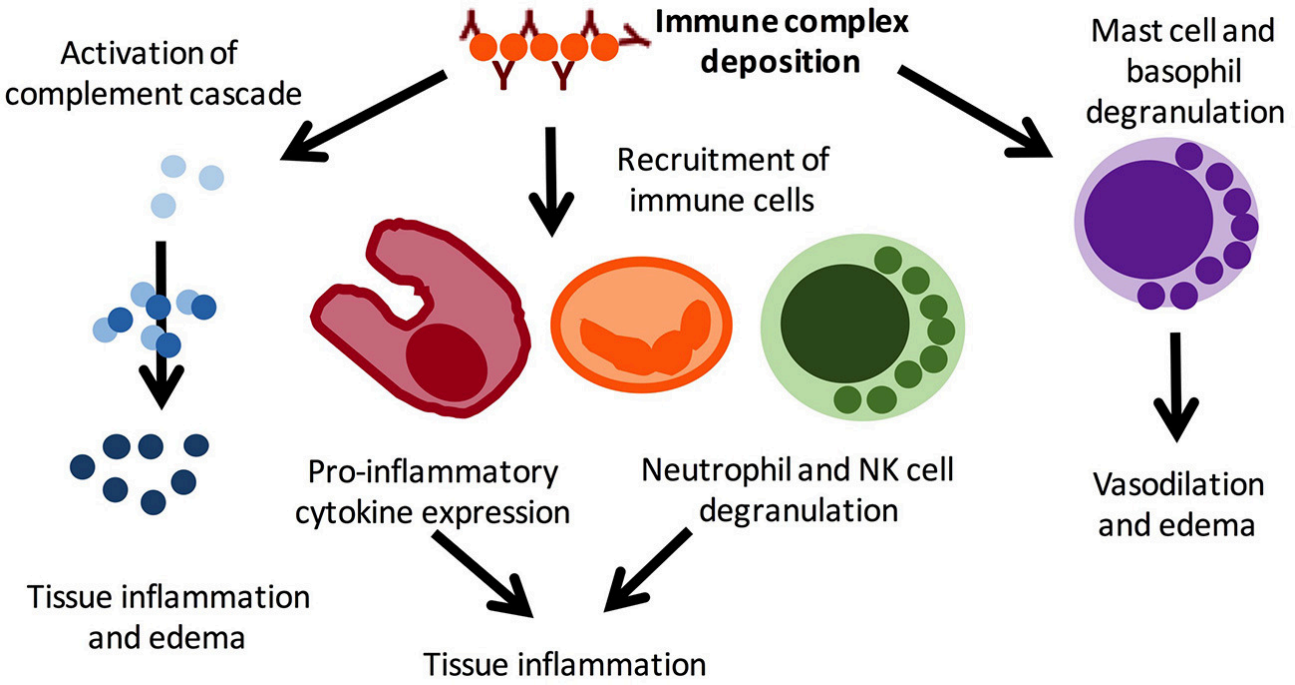
Pro-inflammatory mechanisms in immune complex vasculitis. Immune complex vasculitis is not disease specific and can be a feature or leading symptom of various disorders, including infections and autoimmune/inflammatory conditions. Immune complex deposition result in complement activation, which in turn mediates local inflammation and oedema. This results in the recruitment of immune cells, including macrophages, neutrophils, and NK cells, which further contribute to inflammation and tissue damage through inflammatory cytokine expression. Mast cell and basophil degranulation further amplifying tissue edema and mediates vasodilation. Reproduced with permission from (19).

Cutaneous vasculitis in JSLE is most commonly an immune-complex mediated small-vessel vasculitis (18) (Figure 2). Histological examination of lesions allows determination of the size of vessel affected and immune cells driving inflammation. Typical findings in lupus-related cutaneous vasculitis are small (predominantly) and medium vessel (less commonly) neutrophilic vasculitis with IgG, IgM and/or complement deposition at the basement membrane zone on direct immunofluorescence examination (14).

### Urticarial Vasculitis

Urticarial vasculitis is a recognized rare presentation of SLE presenting with hives lasting more than 24 h which may be entirely asymptomatic, pruritic, or painful. It usually resolves with hyperpigmentation or purpura (18, 20, 21). The incidence of JSLE-associated urticarial vasculitis is unknown but there are several case reports (22–25). Urticarial vasculitis is an immune-complex mediated small-vessel process with leukocytoplastic changes on histology (14). The term “hypocomplementemic urticarial vasculitis” describes the coexistence of hypocomplementemia. Affected individuals frequently exhibit anti-C1q antibodies, which may contribute to altered immune complex processing and removal, and associated systemic involvement (21, 26, 27).

### Cryoglobulinaemic Vasculitis

Cutaneous vasculitis can present with cryoglobulinaemic vasculitis, manifesting as purpuric lesions. IgM and C3 containing immune complexes are present on direct immunofluorescence (Figure 2). Reports on cryoglobulinaemia and vasculitis in children are limited (13, 28).

### Diagnosis

The diagnosis of SLE-associated cutaneous vasculitis is based on clinical assessment. However, where practical and in cases of uncertainty, biopsies should be taken to confirm the diagnosis (15). This is of particular importance because cutaneous vasculitis can be a sign of high disease activity, and may trigger escalation of treatment (3). A study in aSLE patients found that while 36% of patients with digital lesions were clinically diagnosed with vasculitis, following dermatological/histological review only 4% actually had confirmed vasculitis (29). Timing, location, and appropriate depth of biopsy are important for diagnostic accuracy. Ideally, biopsies should be taken within 48 h of the lesions appearing, to avoid false negative results (14).

### Treatment

Topical and low-dose systemic corticosteroids, and/or antimalarial agents (usually Hydroxychloroquine) are usually considered first-line treatment for cutaneous vasculitis. More severe disease may require high-dose steroids, intravenous immunoglobulins (IVIG), plasmapheresis, and/or cytotoxic treatments (18). Rituximab can be effective in some SLE patients with otherwise treatment refractory cutaneous vasculitis (15).

## Visceral Vasculitis

Visceral vasculitis is present in approximately 6% of aSLE patients (30). Reliable data on the prevalence in JSLE does not exist. It can affect a number of organs including the central nervous system (CNS), peripheral nervous system (PNS), lungs, gut, and more rarely the kidneys, heart, and large abdominal and/or thoracic vessels (1). Visceral vasculitis usually manifests in the context of disease flares, and can coexist with cutaneous vasculitis (1).

### CNS Vasculitis

In patients developing neuropsychiatric manifestations of SLE, CNS vasculitis should be considered. However, the contribution of CNS vasculitis in CNS lupus may be limited (1). Indeed, other mechanisms including T-cell and autoantibody-mediated damage to neuronal tissue underlie the majority of neuropsychiatric manifestations (31, 32). Historic postmortem studies estimated the incidence of CNS vasculitis in SLE to be around 7–10% (33). In the context of neuropsychiatric SLE it is particularly important to consider the contribution of antiphospholipid syndrome (APS) (34). SLE vasculitis and APS are the result of very different underlying pathologies that may require differential treatment. APS leads to thrombo-occlusive vasculopathy, warranting treatment with anticoagulation (e.g., heparin) and/or anti-aggregation (e.g., apsirin, clopidogrel) and/or intensive immunosuppressive therapy (e.g., corticosteroids, IVIG, rituximab or other immunosuppressive therapy, plasma exchange) in the context of catastrophic APS (35). Case management is informed by the overall clinical picture and laboratory parameters. While APS is characterized by the presence of persistent moderate-high titers of antiphospholipid antibodies associated with APS, SLE flares with vasculitis are associated with leukopenia, anti-ds-DNA antibodies and hypocomplementaemia (1).

Diagnosing CNS vasculitis requires a high index of suspicion, a systematic multi-disciplinary approach to diagnostic evaluation with MRI imaging playing a central role, and thorough exclusion of the differential diagnoses. Investigations should be directed at the exclusion of underlying conditions, including infections (the most common causes of secondary CNS vasculitis), drug-induced vasculitis, malignancy-associated vasculitis, non-vasculitic inflammatory brain diseases, demyelinating disorders, and antibody-mediated inflammatory brain diseases (36). The recent European evidence-based recommendations for diagnosis and treatment of JSLE (the SHARE initiative) imply that the initial diagnostic work-up of patients with suspected neuropsychiatric JSLE should be performed as in patients without SLE, and lumbar puncture/cerebrospinal fluid analysis, electroencephalogram, neuropsychological assessment of cognitive function, ophthalmologist review, nerve conductional studies, and MRI scanning should be considered. However, SHARE did not provide specific recommendations for suspected SLE CNS vasculitis, but highlighted that a MRI scan cannot exclude neuropsychiatric lupus (37). Even with angiography, false negative results are possible if the small vessels are predominantly involved (38), and brain biopsy may be required (1, 36, 39). Treatment usually involves high-dose glucocorticoids and cyclophosphamide, and may include plasmapheresis and IVIG (40).

### Peripheral Nervous System (PNS) Involvement

Peripheral neuropathies including mononeuritis multiplex can be the result of vasculitis and ischemic damage in JSLE. Mononeuritis multiplex is substantially more common in aSLE, when compared to JSLE patients. It is characterized clinically by symmetric, mild-to-moderately severe sensorimotor polyneuropathy (39). Nerve biopsies usually unveil axonal degeneration and/or depletion, which can be associated with nonspecific vascular changes or chronic perivascular inflammation. Occasionally, biopsies may show necrotising vasculitis (41). As few as two cases of mononeuritis multiplex have been described in JSLE patients to date (42, 43). Other causes of mononeuritis multiplex in children include Eosinophilic Granulomatosis with Polyangiitis (EGPA; formerly known as Churg-Strauss syndrome), polyarteritis nodosa, hypersensitivity/toxic vasculitides, diabetes mellitus, and Tangier's disease (an inborn error of metabolism) (43). Treatment of PNS vasculitis in JSLE may involve high-dose corticosteroids and cyclophosphamide, but is based upon aSLE case series (43).

### Pulmonary Vasculitis

**Figure 3:**
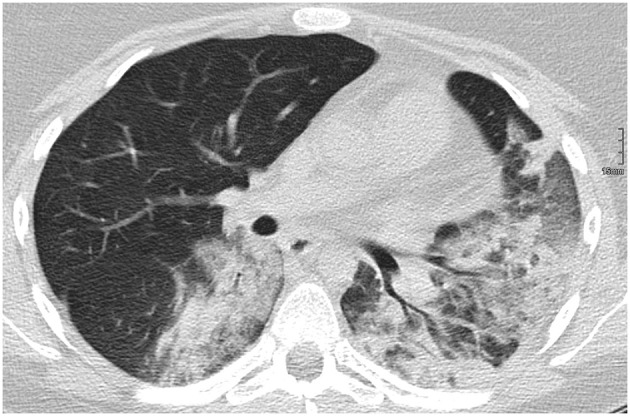
Diffuse alveolar hemorrhage in a 16-year-old SLE patient.

Acute lupus pneumonitis and diffuse alveolar hemorrhage (DAH) are the two most common pulmonary presentations of vasculitis in SLE. Fortunately, both are relatively rare [pneumonitis: 0–14% (44), DAH: 2–5.4% of aSLE patients (45, 46)]. Reports on the prevalence in JSLE currently do not exist. Both conditions may present with sudden-onset dyspnea, cough, fever, and hypoxia. Main clinical differences are pleuritic chest pain in acute lupus pneumonitis, and blood stained sputum or haemoptysis in DAH (47). However, none of these necessarily have to be present. Radiological features of acute lupus pneumonitis on computed tomography (CT) scan include an often bilateral diffuse acinar filling pattern in the lower lobes, and frequently coexists with pleural effusion in ~50% of patients (48). In DAH, diffuse alveolar opacities are characteristically seen on CT (Figure 3). Diffusion capacity for carbon monoxide (DLCO) is usually increased due to the increased availability of hemoglobin within the alveoli (47). In both conditions, bronchoscopy and bronchial alveolar lavage (BAL) are required (where the patient's condition allows it) to exclude infectious differential diagnoses or confirm the diagnosis. In DAH, aspirated fluid is bloody and microscopically red blood cells and haemosiderin-laden macrophages are present (45). Occasionally, an open lung biopsy may be needed to reach a diagnosis of acute lupus pneumonitis, with pathological findings including capillaritis, diffuse alveolar damage and necrosis, cellular infiltrates, hyaline membranes, and sometimes alveolar hemorrhage (47). The pathogenesis of both acute pulmonary pneumonitis and DAH is incompletely understood, but thought to involve deposition of immune complexes in the alveolar septae and blood vessels, and activation of complement leading to capillaritis, with immunohistological studies demonstrating immune complexes which include anti-ds-DNA antibodies and C3 (49) (Figure 2).

Treatment of both acute lupus pneumonitis and DAH is based upon case reports and small case series. Early detection and treatment initiation are crucial. Patients are usually treated with broad-spectrum antibiotics, high-dose corticosteroids and/or cyclophosphamide, and may need mechanical ventilation, IVIG and potentially plasma exchange (46, 47, 50). Both conditions are characterized by poor prognosis with associated mortality rates of ~50%. In those who survive, persistent interstitial infiltrates and lung function abnormalities are frequently seen, with potential for progression on to chronic interstitial pneumonitis (48).

### Gastrointestinal Vasculitis

Gastrointestinal symptoms are not uncommon in JSLE patients and may relate to either treatment-related side effects, infections or JSLE disease activity. Lupus mesenteric vasculitis (LMV), characteristically presents with the clinical picture of an “acute abdomen” with sudden onset, diffuse and severe abdominal pain which can be associated with nausea, rectal bleeding, and vomiting (51). Of note, corticosteroids and/or other immunosuppressive treatments may mask symptoms and result in diagnostic and therapeutic delay. The estimated prevalence of LMV ranges between 0.2 and 9.7% among aSLE patients, making up for ~29–65% of aSLE patients presenting with an acute abdomen (51). In a Taiwanese study comparing aSLE and JSLE patients presenting with acute abdominal pain, LMV was the most common cause of admission in the JSLE patients. Furthermore, LMV was significantly more prevalent in JSLE patients when compared to the aSLE group [12/38 (31.6%) vs. 15/1081 (3.9%); *p* = 0.016], occurring in JSLE patients with high disease activity (SLE disease activity index, SLEDAI scores >8). Children were also found to be more likely to experience recurrent episodes of LMV (39.1% vs. 14.8%; *p* = 0.009) (52). In contrast to this study, other studies have reported LMV in JSLE patients with low SLEDAI scores (53, 54).

Bowel ischemia secondary to LMV can result in perforation, hemorrhage and high mortality rates of up to 50%. The importance of early laparotomy was emphasized by a study demonstrating significantly higher survival in patients who underwent early intervention (0/33 deaths when laparotomy was undertaken within 24–48 h vs. 10/11 when laparotomy was performed after 48 h) (55). LMV most frequently affects the superior mesenteric artery that supplies the ileum and jejunum (80–85%), with the rectum less frequently affected (14%), and gastric involvement being very uncommon (56). Abdominal CT is a useful investigation for diagnosing LMV. It is characterized by the presence of dilated bowels, target lesions (abnormal bowel wall enhancement), comb signs (engorgement of mesenteric vessels), bowel wall thickening, and ascites (57). Histology may demonstrate arteritis, venulitis, immune complex, C3, and fibrinogen deposition, inflammatory cell infiltration, necrosis, and thrombosis of vessels affected (58). No randomized trials are available investigating treatment and associated outcomes in LMV. Currently, treatment involves “bowel rest,” intravenous corticosteroids, and cyclophosphamide in severe cases (39, 53, 58, 59). Successful use of Rituximab has also been described in case series (58).

### Renal Vasculitis

The prevalence of renal vasculitis in JSLE has not been investigated. In aSLE, vasculitic changes affecting the larger arterioles and small kidney arteries may (rarely) accompany proliferative lupus nephritis (60). Focal segmental necrotizing glomerulonephritis with fibrinoid necrosis can be seen, and may lead to rapidly progressive renal failure (61). In a Spanish study evaluating the prevalence and clinical characteristics of vasculitis in a cohort of 670 aSLE patients, 76/670 (11%) patients exhibited any vasculitis, but “only” 2/76 (3%) experienced renal vasculitis (13). Treatment of renal vasculitis in JSLE usually involves high-dose corticosteroid and cyclophosphamide (39).

### Cardiac Vasculitis

Cardiac involvement in JSLE typically comprises pericarditis, cardiomegaly, valvulitis, and conduction abnormalities. SLE is associated with an increase in coronary heart disease risk, and a 50-fold increased risk of myocardial infarction (62). Coronary artery vasculitis occurs in both JSLE and adult SLE, but is rare across all age groups (63–66). Clinical differentiation of “classical” coronary artery disease from coronary arteritis is difficult and requires angiography. Coronary vasculitis usually results in segments of tapered narrowing, coronary ectasia, and/or aneurysms. Histopathological examination reveals thrombosis, deposition of immune complexes, infiltration of lymphocytes/neutrophils, and associated fibrinoid necrosis (64). A retrospective study assessed findings and outcomes in JSLE patients who were referred for echocardiography during their initial presentation for either tachycardia or a new murmur. Four patients demonstrated coronary artery dilatation (suggestive of coronary arteritis) which resolved once JSLE was treated, leading the authors to conclude that coronary arteritis may be more common in JSLE than previously appreciated (63). In a retrospective Taiwanese study looking at cardiopulmonary involvement in JSLE over a 20-year period, 6/157 patients exhibited coronary artery abnormalities, including vascular dilation, aneurysms, vasculitis, and stenosis (67). With SLE itself being a risk factor for cardiovascular disease, further studies are required longitudinally evaluating the impact of echocardiogram surveillance and treatment of coronary vasculitis on long-term cardiovascular risk.

### Aortic Vasculitis

Vasculitis in SLE predominantly affects medium and small vessels. Aortic (large vessel) involvement has been reported in small case series and collated within a meta-analysis of 35 cases, of which 5/35 patients developed SLE in childhood, with aortic involvement manifesting between the ages of 23 and 38 years old. Thoracic aneurysms correlated with dissection and cystic medial degeneration, while abdominal lesions correlated with atherosclerosis. A total of 21/35 (60%) cases required surgery and death was observed in 11/35 (31.4%) patients. Thoracic lesions resulted in higher mortality rates than abdominal lesions (68). Treatment of aortic vasculitis includes corticosteroids, cyclophosphamide and blood pressure control. Surgery may be required in some cases (39, 69). See Table 1 for a summary of the prevalence of different vasculitic manifestations and the main treatments used.

**Table 1:** Prevalence and treatment of different vasculitc manifestations in JSLE.

**Vasculitic manifestation**	**Prevalence in JSLE**	**Usual treatments**
Cutaneous vasculitis	12–21.6% (7, 8)	• Mild—moderate disease/first line treatments—topical and low-dose systemic corticosteroids, and/or antimalarial agents. • More severe disease/second line treatment—high-dose steroids, IVIG, plasmapheresis and/or cytotoxic treatments (18). Rituximab is an option with otherwise treatment refractory cutaneous vasculitis (15).
CNS vasculitis	NA	High-dose glucocorticoids and cyclophosphamide, and may require plasmapheresis and IVIG (40).
PNS vasculitis	NA	High-dose corticosteroids and cyclophosphamide (43).
Pulmonary vasculitis	NA	Broad-spectrum antibiotics, high-dose corticosteroids, and/or cyclophosphamide. May need mechanical ventilation, IVIG, and potentially plasma exchange (46, 47, 50).
Gastrointestinal vasculitis	31.6%^*^ (52)	Bowel rest, intravenous corticosteroids, and cyclophosphamide in severe cases (39, 53, 58, 59). Use of Rituximab has also been described in case series (58).
Aortic vasculitis	NA	Corticosteroids, cyclophosphamide, and blood pressure control. Surgery may be required in some cases (39, 69).

* *31.6% of all JSLE patients presenting with an acute abdomen (52). JSLE, juvenile systemic lupus erythematosus; NA, not available; IVIG, intravenous immunoglobulin*.

## SLE Vasculitis vs. Anti-phospholipid Syndrome

In aSLE patients, APS is associated with cutaneous vasculitis (30, 70). Differentiating between vasculopathy/thrombosis and vasculitis in SLE-associated APS can be difficult. However, it is of utmost importance as treatment approaches are different (anti-inflammatory treatment for vasculitis vs. anti-thrombotic therapy for vasculopathy) (71). As mentioned above, histological assessment of cutaneous lesions can differentiate vasculitis from cutaneous manifestations of APS. This is particularly relevant in the context of ischaemic lesions which are difficult to differentiate clinically. Where biopsy is not practical, it is important to consider the overall context of the presentation and potential evidence of other APS-related manifestations such as thrombosis. In cases with extra-cutaneous evidence of SLE disease flare or systemic vasculitis, immunosuppressive treatment should be favored (13).

## Vasculitis in the Context of “SLE-Like” Disease

Primary type-I interferonopathies are a group of Mendelian disorders that share the upregulation of type-I interferon signaling as key pathophysiological feature. These monogenic diseases include (but are not limited to) Aicardi-Goutières syndrome and syndromic forms of SLE-like disease (72, 73). A common clinical feature across type I interferonopathies is skin involvement with chilblain lesions and/or vasculitis as well as systemic vasculopathy and/or vasculitis, all of which can also been seen in patients with “classical” JSLE. Though very rare and not fully reflecting the molecular and clinical phenotype of JSLE, primary type I interferonopathies and their pathophysiology are useful “models” for “classical” JSLE, which is also frequently characterized by type I interferon activation. Furthermore, a number of other SLE-like conditions, including complement deficiencies, are characterized by type I interferon responses that are not primarily caused by mutations in positive or negative regulators of interferons, but the result of immune complex accumulation, another key contributor to the pathophysiology of SLE (74, 75).

Aicardi-Goutières syndrome manifests with progressive encephalopathy that is associated with calcification of the basal ganglia, mimicking congenital viral infections (76). Clinical skin manifestations include chilblain lesions, which are the name giving feature of familial chilblain Lupus, another monogenic SLE-like disorder characterized by type I interferon upregulation (Figures 1D,E). Aicardi-Goutières syndrome, familial chilblain Lupus and other type I interferonopathies demonstrate vasculopathy, immune complex deposition, and lymphocytic vasculitis on skin biopsy (73, 77). Indeed, in severe cases peripheral vasculopathy can result in atrophy and even digital auto-amputation in some patients (Figure 1F) (72). This has particularly been reported in spondyloenchondrodysplasia with immune dysregulation (SPENCDI), a rare immuno-osseous dysplasia caused by biallelic mutations in the *ACP5* gene (72) and Stimulator of interferon genes (STING)-associated vasculopathy with onset in infancy (SAVI) that includes heterozygous mutations in *TMEM173* (78). Both disorders, in addition to increased activation of type I interferon responses, share clinical features with “classical” SLE including neurological and immune manifestations in SPENCDI, and pyrexia, vasculitis, microthrombotic angiopathy, and interstitial lung disease in SAVI (78).

## Conclusions

JSLE is a rare, heterogeneous and complex condition. This translates to difficulty in the recognition and management of disease, particularly when less common manifestations, such as vasculitis are involved. Cutaneous is more common than visceral vasculitis, and more prevalent in JSLE when compared to adult-onset disease. Reports on visceral vasculitis in JSLE are limited, which may be a reflection of difficulty in achieving the diagnosis and differentiating vasculitis from other JSLE-related complications. Early recognition and treatment of SLE-associated vasculitis are paramount to optimizing outcomes and preventing tissue and organ damage. Collaborative approaches are required to improve our knowledge on the demographics, clinical presentations, disease courses, and treatment options in JSLE-associated vasculitis.

## Author Contributions

ES, HL, and CH all participated in review and interpretation of the literature. All authors were involved in drafting the manuscript and revising it critically for important intellectual content. They have also all read and given final approval of the version to be published.

### Conflict of Interest Statement

CH's work is supported by the Fritz-Thyssen Foundation (research grant: SLE), Novartis Pharmaceuticals (research grant: psoriasis), LUPUS UK (research grant: SLE), the Hugh Greenwood Legacy Fund (research grant: bronchial inflammation), the Michael Davie Research Foundation (research grant: CNO/CRMO), and the FAIR charity (research grants: bronchial inflammation and SLE). CH received honoraria for advisory board activities and presentations from Novartis pharmaceuticals and Roche (systemic autoinflammatory disease). The remaining authors declare that the research was conducted in the absence of any commercial or financial relationships that could be construed as a potential conflict of interest.
